# Paraspinal Muscle Changes in Individuals with and without Chronic Low Back Pain over a 4-Month Period: A Longitudinal MRI Study

**DOI:** 10.3390/medicina60030490

**Published:** 2024-03-16

**Authors:** Meagan Anstruther, Monica Sean, Pascal Tétreault, Maryse Fortin

**Affiliations:** 1 Department Health Kinesiology and Applied Physiology, Concordia University, Montreal, QC H4B 1R6, Canada; 2 Faculty of Medicine and Health Sciences, Université de Sherbrooke, Sherbrooke, QC J1H 5N4, Canada; monica.sean@usherbrooke.ca (M.S.); pascal.tetreault@usherbrooke.ca (P.T.); 3 Department of Anesthesiology, Université de Sherbrooke, Sherbrooke, QC J1H 5N4, Canada; 4 Centre de Recherche du CHUS, Sherbrooke, QC J1H 5N4, Canada; 5 Department of Nuclear Medicine and Radiobiology, Université de Sherbrooke, Sherbrooke, QC J1H 5N4, Canada; 6 School of Health, Concordia University, Montreal, QC H4B 1R6, Canada; 7 Centre de Recherche Interdisciplinaire en Réadaptation (CRIR), Montreal, QC H3S 1M9, Canada

**Keywords:** low back pain, imaging, paraspinal musculature, fat infiltration

## Abstract

*Background and Objectives*: Previous research has shown associations between atrophy and fatty infiltration of the lumbar paraspinal musculature and low back pain (LBP). However, few studies have examined longitudinal changes in healthy controls and individuals with LBP without intervention. We aimed to investigate the natural variations in lumbar paraspinal musculature morphology and composition in this population over a 4-month period. *Materials and Methods*: Healthy controls and individuals with LBP were age- and sex-matched and completed several self-administered questionnaires. MRIs of L1–L5 were taken at baseline, 2 months, and 4 months to investigate cross-sectional area (CSA), along with DIXON fat and water images. A total of 29 participants had clear images for at least one level for all three time points. Means and standard deviations were calculated for the participant demographics. A two-way repeated measures ANOVA was performed to investigate CSA, fat signal fraction, and CSA asymmetry. *Results*: A total of 27 images at L3/L4, 28 images at L4/L5, and 15 images at L5/S1 were included in the final analysis. There were significant main effects of group for psoas CSA at the L3/L4 level (*p* = 0.02) and erector spinae (ES) CSA % asymmetry at the L3/L4 level (*p* < 0.001). There was a significant main effect of time for lumbar multifidus (LM) CSA % asymmetry at L4/L5 level (*p* = 0.03). *Conclusions*: This study provides insights into LM, ES, and psoas morphology in both healthy controls and affected individuals over a 4-month period without any intervention. Our findings suggest that psoas CSA at higher lumbar levels and CSA % asymmetry in general may be a better indicator of pathology and the development of pathology over time. Evaluating natural variations in paraspinal musculature over longer time frames may provide information on subtle changes in healthy controls and affected individuals and their potential role in chronic LBP.

## 1. Introduction

Low back pain (LBP) continues to be highly prevalent across the globe and is the number one cause of disability, affecting people of all ages, income statuses, and regions [1]. LBP is defined as pain occurring between the lower rib and gluteal fold, with or without neurological symptoms [1]. Chronic LBP can be defined as pain every day for 3 months or pain for at least half the days in the last 6 months [1,2]. Many studies have examined contributing factors to LBP, such as genetics, psychological factors, societal status, and physiological factors. However, there is no singular cause of LBP, making it difficult not only to study but also to manage [1,3,4,5,6,7]. For physiological factors, lumbar paraspinal musculature has recently been a major focus in LBP research. Studies have shown the benefit of targeting deep trunk and paraspinal muscles with exercise programs to reduce pain and atrophy [7]. However, the current understanding of the role of paraspinal musculature in the development, recurrence, and severity of LBP remains limited, especially when it comes to the natural progression (i.e., without intervention) of the paraspinal muscles’ structural changes over time.

Given the importance of the lumbar paraspinal musculature and its critical role in spinal stability, the associations between paraspinal muscle structural and functional changes and LBP has received increased attention in the last decades. However, the majority of human studies are cross-sectional in nature or include some form of intervention (i.e., therapeutic exercise, etc.) [7]. A recent meta-analysis found atrophy and increased fatty infiltration of the lumbar multifidus (LM) muscle in individuals with LBP, while the erector spinae (ES) and psoas major (PS) did not present with significant changes [4]. However, there are still inconsistencies within the literature. While many studies have reported atrophy of the LM, some have found no change and even hypertrophy, in addition to reporting increased LM fat infiltration [5,6,7,8]. Furthermore, the literature on the ES and other paraspinal muscles is very scarce [7]. While LM asymmetry >10% was suggested to be associated with spinal pathology [9], this was also observed in healthy men, in addition to ES asymmetry ranging from 8.2% to 18.8% in a similar healthy population [10]. With most LBP studies being cross-sectional in nature, it is imperative to investigate the degree of natural structural changes that occur in lumbar paraspinal musculature over time, both in healthy and symptomatic individuals, to better understand their role in chronic LBP.

While a few studies explored morphological changes in paraspinal musculature in healthy individuals and those with LBP, we are only aware of three longitudinal studies investigating the natural progression (i.e., without intervention) of paraspinal muscle changes [3,11,12]. One study investigated LM intramuscular adipose tissue in 40-year-olds over a nine-year period, finding some cross-sectional associations between severe and moderate LM intramuscular adipose tissue and the presence of LBP and leg pain but no longitudinal associations [11]. A second study investigated the relationship between the presence of LM intramuscular adipose tissue and the effects of physical activity on developing LBP in children over a 12-year period, finding all associations to be non-significant after adjusting for sex and BMI [12]. A 15-year study on male twins found similar morphological changes in the LM and ES over time. However, no association was found between paraspinal morphology and physical demands or LBP history [3]. It remains unclear whether paraspinal muscle changes occur as a result of LBP or are the cause of LBP. Furthermore, we are unaware of any studies that have assessed short/mid-term temporal changes in paraspinal muscle morphology and composition both in healthy controls and in individuals with LBP without any therapeutic intervention. Thus, this study aimed to investigate and quantify the amount of natural change that occurs in lumbar paraspinal muscle morphology (e.g., size, asymmetry) and composition (e.g., fatty infiltration) in healthy controls and individuals with LBP over a four-month period.

## 2. Materials and Methods

### 2.1. Participants

The current study was a secondary analysis of data from an ongoing study at the Centre hospitalier universitaire de Sherbrooke (CHUS), thus no sample-size calculation was carried out for the current study [13]. To maximize sample size, all subjects with good quality MRI images were included. Individuals with chronic LBP were recruited through posters at the Centre intégré universitaire de santé et de services sociaux de l’Estrie-Centre hospitalier universitaire de Sherbrooke (CIUSSS-CHUS), Facebook ads, and word of mouth. Controls were recruited from the community as a convenience sampling. Inclusion criteria for healthy controls included being a minimum of 18 years old and having no pain from an injury, no history of chronic LBP, and no outstanding painful episode in the previous 3 months. Inclusion criteria for individuals with LBP included being a minimum of 18 years old; being able to read or understand French; suffering from chronic LBP for at least 4 months with no other episode of back pain; having pain intensity of ≥3/10 in the 24 h period before the initial visit; and having no exposure to corticosteroid injection in the last 2 years, no neurological disorder, and no claustrophobia. Both healthy controls and the chronic LBP group were excluded if they suffered from a neurological, cardiovascular, or pulmonary disorder or comorbid pain syndrome; had any surgical intervention in the back; used opioids, antidepressants, anticonvulsants, or psychostimulants; had received recent (<1 year) corticosteroid infiltration; were pregnant or considering becoming pregnant at any time during the study; or had any MRI contraindications. There was no exclusion based on ethnicity or gender. Participants were excluded if they had a previous history of invasive or aggressive treatment to manage their pain. Healthy controls and individuals with LBP were age- and sex-matched in the CHUS study. From the original cohort taking part in the CHUS study, all 52 participants were eligible for the current study. Ethics approval was granted from the institutional review board of the CIUSSS-CHUS (Sherbrooke, QC, Canada; approval #2021-3861). All participants provided informed consent.

### 2.2. Design

Participants had three visits: baseline, 2 months, and 4 months. There was no treatment provided except for what patients were already taking and no requirement to discontinue any medication. MRI imaging and questionnaires were completed at each visit.

### 2.3. Questionnaires

Participants from the original cohort in the CIUSSS-CHUS study completed several self-administered questionnaires, including the Pain Catastrophizing Scale (PCS), painDETECT, Brief Pain Inventory (BPI), Central Sensitization Inventory (CSI), Pain Disability Index (PDI), McGill Pain Questionnaire (MPQ), State-Trait Anxiety Inventory (STAI), and Pain Outcomes Questionnaire (POQ). Healthy controls only completed the PCS and STAI. For the purposes of the current study, only the PCS [14,15], painDETECT [16], BPI [17], CSI [18,19], and PDI [20,21] were included in the demographic analyses. All questionnaires used in the current study were validated in French. 

### 2.4. MRI Imaging

Using a 3Tesla MRI (Philips Healthcare, Best, The Netherlands), axial T2-weighted and DIXON scans (matrix: 312 × 312 × 32 slices; FOV: 250 mm × 250 mm × 128 mm; voxel: 0.8 mm × 0.8 mm × 4 mm) were collected from L3/L4 to L5/S1. The images were stored offline.

### 2.5. Paraspinal Muscle Measurements

**Figure 1:**
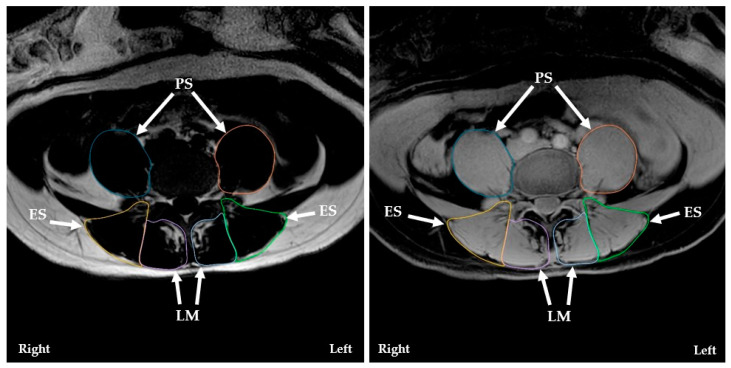
Fat (**left**) and water (**right**) images of lumbar multifidus (LM), erector spinae (ES), and psoas (PS) at the L4/L5 level.

The LM, ES, and PS were measured at L3/L4, L4/L5, and L5/S1, except for the PS at the L5/S1 level due to images being cut off. Horos (v4.0.0, Geneva, Switzerland) was used for all measurements, as well as the reconstruction of images, to ensure a mid-disc slice. To measure the cross-sectional area (CSA) of each muscle, the borders were traced on right and left sides on the fat DIXON images (Figure 1), following the recommendations from Hodges et al. (2021) [22]. Measurements were then copied onto their respective water DIXON images. The fat signal fraction (FSF) for the right and left sides of each muscle was calculated using the following equation: [signal fat/(signal water + signal fat)] × 100%. Relative CSA asymmetry was calculated for each muscle using the following formula: [(larger side − smaller side)/larger side] × 100%.

### 2.6. Statistical Analysis

Means and standard deviation were calculated for participant demographics and pain questionnaires using SPSS (IBM SPSS Statistics, New York, NY, USA, v.29.0.0.0). Independent *t*-tests and chi-square tests were used to compare continuous and categorical demographic variables between groups, respectively. A two-way repeated measures ANOVA was completed for the PCS. One-way repeated measures ANOVA were performed for the remaining pain questionnaires. Means were calculated for paraspinal muscle measurements of interest (e.g., CSA and FSF). Two-way repeated measures ANOVA of CSA, FSF, and CSA asymmetry were performed for the LM, ES, and PS at L3/L4 and L4/L5 and for the LM and ES at L5/S1, adjusting for BMI. The normality and sphericity assumptions were verified and met for each muscle of interest, except PS CSA at the L4/L5 level, where a Friedman test was performed instead. A *p*-value of <0.05 and CI of 95% was used for all statistical analysis.

## 3. Results

A total of 29 participants (16 control (5 male, 11 female), 13 LBP (7 male, 6 female)) had clear images at a minimum of one of the three lumbar levels investigated at all three time points. Due to missing images at lumbar levels and/or time points, poor image quality, and inability to reconstruct images, a total of 27 images at L3/L4, 28 images at L4/L5, and 15 images at L5/S1 were included in the final analyses. Participants’ demographic characteristics are presented in Table 1. Mean age was 39.19 ± 13.01 years and 41.77 ± 13.71 years for the controls and LBP group, respectively. The control group was 31.3% male and 68.7% female, while the LBP group was 53.8% male and 46.2% female. Mean BMI was 23.71 ± 2.78 and 27.80 ± 4.82 for the control and LBP groups, respectively. All participants in the LBP group had pain lasting longer than 6 months. Pain questionnaire scores completed by both healthy controls and the LBP group and pain questionnaire scores completed by the LBP group only are presented in Table 2a and Table 2b, respectively. PCS questionnaires were completed by all 29 participants with a significant main effect of time between baseline and 2 months, and between baseline and 4 months (both *p* < 0.001). There was also a significant main effect of group and as a significant time × group interaction. For the pain questionnaires completed solely by the LBP group, PDI, BPIi, and BPIs had significant main effects of time. The LBP group had more diversity in ethnicity compared to the control group, which was 93.8% Caucasian. The control group was also only comprised of individuals who had a college or university educational level. In the LBP group, 53.8% had an annual income of <$50 000, compared to 31.3% in the control group.

### 3.1. CSA Measurements

The results of the CSA measurements are presented in Table 3. There was a significant negative difference between 2 months and 4 months for PS CSA at the L4/L5 level (*p* < 0.05) in the control group. There were no significant interactions between time and group or main effect of time or group for any muscle or level, except for a significant main effect of group for PS CSA at the L3/L4 level.

### 3.2. FSF Measurements

The results of the FSF measurements are presented in Table 4. There was a significant positive difference in ES FSF at L4/L5 between 2 months and 4 months (*p* < 0.05) and a significant negative difference in ES FSF at L5/S1 between baseline and 2 months (*p* < 0.05) in the control group. There were no significant interactions between time and group or main effect of time or group for any muscle or level.

### 3.3. CSA % Asymmetry Measurements

The results of the CSA % asymmetry measurements are presented in Table 5. There was a significant increase in LM CSA % asymmetry at the L4/L5 level between baseline and 2 months (*p* = 0.01) in the LBP group, and a significant main effect of group for ES CSA % asymmetry at the L3/L4 level (*p* < 0.001). There was a significant main effect of time for LM CSA % asymmetry at the L4/L5 level (*p* = 0.03). There were no other significant interactions or main effects of time or group.

## 4. Discussion

Overall, there were no significant difference or main effects of time or group nor interactions (e.g., time × group) for CSA or FSF for any of the three muscles at all levels investigated, except for a significant main effect of group for PS CSA at the L3/L4 level. Previous studies have reported comparable CSA values in individuals with LBP at L4 (LM: male 11.5 cm^2^, female 9.3 cm^2^; ES: male 19.2 cm^2^, female 15.9 cm^2^ [23]) and the L3/L4 level (LM: baseline 7.21 cm^2^, 15-yr post 7.09 cm^2^; ES: baseline 20.45 cm^2^, 15-yr post 20.15 cm^2^ [3]) compared to our study (LM: 9.5–9.7 cm^2^, ES: 18.1–18.3 cm^2^). However, one study reported larger FSF values (LM: 45.0–52.0%; ES: 42.0–44.0%) compared to the current study (LM: 28.5–28.8%; ES: 31.9–33.0%), which may be a result of measurement differences, the large sample size, or that participants were candidates for surgery (i.e., more variety of participants and conditions than just individuals with non-specific LBP were included) in the previous study [23]. Interestingly, LM atrophy and increased fat infiltration, as well as increased fat infiltration in the ES but to a lesser extent than the LM, have been commonly observed in previous research [7]. However, the majority of studies are cross-sectional, examining biopsies or images from a single time point, thus potentially missing subtle changes that may occur in healthy and affected lumbar paraspinal musculature morphology and composition as time progresses. The PS muscle is not often investigated in the LBP population despite its anatomical attachments to the lumbar spine [4,5]. Our findings suggest that PS CSA towards the upper levels, where the PS attaches onto the front of the lumbar spine, was significantly larger in the LBP group across all time points. It may be that a larger and potentially stronger PS could pull on the lumbar segments, leading to changes in spinal stability that may affect observed pain levels, especially if the LM is not strong enough to stabilize against these forces. It would be interesting to investigate the PS at the L1/L2 and L2/L3 levels to see if there are any other differences in individuals with LBP at the other attachment points to the spine.

In contrast to CSA and FSF, there were significant main effects of time for LM asymmetry at L4/L5 and group for ES asymmetry at L3/L4. Previous studies have found a threshold of 8–10% in LM CSA asymmetry to be related to the presence of LBP in the general population [9]. However, another study found >10% LM CSA asymmetry and 8.2–18.8% ES CSA asymmetry in healthy males [10]. Our findings revealed both healthy and LBP individuals had an average of <9% LM CSA asymmetry at all levels. While the ES also had an average of <9% CSA asymmetry at L3/L4 and L4/L5, there was a significant main effect of group at the L3/L4 level, with the LBP group having greater asymmetry. Interestingly, at L5/S1, ES CSA asymmetry ranged from 17.7–20.4% in both healthy and LBP groups. This large change in CSA asymmetry was only observed in the ES at L5/S1 and in no other muscle or level. While our study did not separate left and right sides to compare muscle morphology and composition, it would be interesting to see if the smaller side of the ES at L5 had any correlation with the smaller side in the LM and PS. Previous research suggested that LM CSA % asymmetry at L5 was the best predictor of lower limb injury in athletes [24]. With increased asymmetry of the LM, one may expect the ES to be smaller on the contralateral side as a means of compensation to aid in stabilization of the spine on the affected side. There may also be the possibility that the ES becomes smaller on the ipsilateral side due to potential decreases in innervation to the affected area. However, a larger sample would be needed to provide more meaningful results. To our knowledge, this is the first time CSA % asymmetry was measured in the PS, ranging from 4.7–8.2% in healthy individuals and 6.3–9.6% in individuals with LBP. Our findings suggest that CSA % asymmetry may be a better indicator of pathology over time and should be included in future longitudinal studies.

Our results corroborate with some longitudinal studies investigating paraspinal musculature and LBP. Hebert et al. investigated LM intramuscular adipose tissue and reported no significant relationship between the presence of intramuscular fat in the LM and the presence of LBP over time after adjusting for sex, BMI, and leisure activity [11]. However, the participants at time 1 (40 years old) with severe LM intramuscular adipose tissue had greater odds of experiencing previous LBP, nontrivial LBP, or leg pain [11]. Another study in children also found that participants with high LM intramuscular adipose tissue had increased odds of developing LBP, yet these results became non-significant after adjusting for sex and BMI [12]. In our study, FSF did not have any significant interactions over time, and only showed significant differences between time points in the ES. This indicates that, while intramuscular fat may play a role in increased odds of developing LBP, our findings suggest that paraspinal muscle composition (e.g., FSF) remained consistent over a 4-month period in individuals with chronic LBP. A 15-year longitudinal study found a decrease in LM and ES CSA, an increase in fatty infiltration, and increased CSA % asymmetry over time [3]. They found that spinal level was a major influence compared to age and that paraspinal muscles have similar morphological changes over time, which was also observed in our study [3]. Hodges et al. noted smaller LM and psoas CSA in individuals with chronic LBP and mixed results for ES CSA. However, most studies are cross-sectional in nature, indicating a need for more longitudinal studies to capture the morphological changes in paraspinal musculature over time [7,25]. Previous research has also indicated that LM muscle wasting occurs in individuals with acute unilateral LBP [26], and that the LM does not automatically recover, showing decreased LM CSA even after 10 weeks without intervention [27]. Our results showed improvement in pain over the course of 4 months and the beginnings of morphological changes in paraspinal musculature over a period of months without intervention. All other longitudinal studies were completed over longer time periods (i.e., years) and did reveal changes in paraspinal musculature; thus, longer periods of time may be required to detect changes in paraspinal muscles, or a greater level of disability is also needed. Thus, while LBP may last for years, it is important to investigate what occurs with lumbar paraspinal musculature in the early stages of LBP to better understand the progression over time.

### 4.1. Demographics

The LBP group had a greater diversity of ethnicity and education level, in addition to having a lower annual income when compared to the control group. It has been previously shown that individuals with lower educational levels tend to have lower incomes or manual-labor intensive careers, which are both associated with developing LPB and may explain why a greater number of our LBP group fell into <$50,000 categories [28,29]. The LBP group also had relatively low scores on the PDI (mean <19 points at all visits). The PDI gives an indication of the level of disability attributed to chronic pain, indicating the population included in this study had a level of disability that was not severely affecting their daily activities. This may explain why significant interactions were not observed for groups. If quality of life is not affected greatly, there may not be as large of a difference between CSA, FSF, and CSA % asymmetry between controls and individuals with LBP. However, the current study did not include any specific LBP-related disability questionnaires, such as the Oswestry Disability Index. While the PDI provides information on general chronic pain, it would be beneficial to investigate the specific effects of LBP-related disability in this particular population. While the pain questionnaires were not an objective of this study, it is interesting to note that most values decreased over the 4-month period for the LBP group even without an intervention administered, indicating there may be other factors to consider in future investigations (Table 2) [30].

### 4.2. Limitations

Due to the small sample size, males and females were not separated, which may have affected the observed CSA, FSF levels, and weight differences observed in the demographics, though sex was determined not to be a significant covariate. Another limitation is that only three lumbar levels were investigated, with a small number of images available at the L5/S1 level.

### 4.3. Future Directions

To date, there have been few longitudinal studies investigating paraspinal musculature, mainly focusing on the LM and ES and intramuscular adipose tissue. This study is the first to our knowledge to provide insights into LM, ES, and PS morphology in both healthy controls and individuals with LBP over a 4-month period without any intervention. Future studies with larger sample sizes are needed to confirm and expand our findings and examine differences in the natural changes in paraspinal musculature over time between sexes. In addition, it would be interesting to see how paraspinal musculature changes beyond a 4-month period without intervention, as the muscular properties observed in the current study appear to be mostly stable. This may help to determine subtle changes that occur in both healthy individuals and individuals with LBP as time progresses and may provide insights into the beginning stages of paraspinal muscle changes in individuals who develop chronic LBP.

## 5. Conclusions

Overall, paraspinal musculature CSA and FSF remained relatively stable over a 4-month period without any intervention, both in LBP and healthy control groups. However, our findings suggest that CSA % asymmetry may be a better indicator of pathology over time when investigating subtle changes in paraspinal musculature. Understanding paraspinal muscle changes at different time points and their complex interrelationships with pain mechanisms and functional changes is critical to clarify their implications for rehabilitation.

## Figures and Tables

**Table 1:** Means ± standard deviation for participant demographics and pain questionnaires.

	Controls (*n* = 16; Male = 5, Female = 11)	LBP (*n* = 13; Male = 7, Female = 6)	*p*-Value
**Age**	39.19 ± 13.01	41.77 ± 13.71	0.79
**Height (cm)**	166.81 ± 9.07	170.17 ± 10.66	0.54
**Weight (kg)**	66.13 ± 10.73	81.31 ± 19.41	0.05
**BMI**	23.71 ± 2.78	27.80 ± 4.82	0.15
**Ethnicity**			0.15
Caucasian	15	9	
Asian	1	1	
Hispanic	0	2	
African	0	0	
Middle Eastern	0	1	
**Education Level**			0.06
Primary School	0	0	
High School	0	0	
Apprenticeship	0	3	
College	5	2	
University	11	8	
**Annual Income**			0.04
<20 K	4	1	
20 K < 35 K	1	4	
35 K < 50 K	0	2	
50 K < 65 K	5	4	
65 K < 80 K	4	0	
80 K < 100 K	2	2	
>100 K	0	0	
**Pain Duration**	N/A		
4–5 months		0	
6–12 months		4 (30.8%)	
1–4 years		3 (23.1%)	
5+ years		6 (46.2%)	

**Table 2:** (**a**) Means ± standard deviation for the Pain Catastrophizing Scale (PCS) pain questionnaire. (**b**) Means ± standard deviation for pain questionnaires completed by the LBP group.

**(a)**				
	**Controls (*n* = 16)**	**LBP (*n* = 13)**	**Main Effect of Group**	**Time × Group Interaction**
**PCS**				
Baseline	8.25 ± 8.52	21.69 ± 13.60	*p* = 0.02 F = 6.66 df = 1	*p* = 0.001 F = 7.82 df = 2
2 Months	6.63 ± 7.26	14.00 ± 9.82 *		
4 Months	5.38 ± 7.31	11.31 ± 11.78 ***		
MD (95% CI)	−2.88 (−7.00 to 1.23)	−10.39 (−14.93 to −5.84)		
**Main effect of time**	*p* < 0.001 F = 22.82 df = 2			
**(b)**				
	**LBP (*n* = 13)**	**MD (95% CI)**		**Main Effect of Time**
**PDI**				
Baseline	18.15 ± 8.82	−9.46 (−18.10 to −0.82)		*p* = 0.03 F = 3.90 df = 2
2 Months	14.85 ± 9.11			
4 Months	8.69 ± 8.37			
**BPIi**				
Baseline	20.54 ± 9.49	−11.31 (−20.04 to −2.57)		*p* = 0.01 F = 5.42 df = 2
2 Months	16.46 ± 9.38			
4 Months	9.23 ± 7.61			
**BPIs**				
Baseline	18.00 ± 3.22	−5.62 (−11.49 to 0.26)		*p* = 0.04 F = 3.59 df = 2
2 Months	17.62 ± 5.62			
4 Months	12.38 ± 8.06			
**painDETECT**				
Baseline	8.46 ± 4.18	−0.46 (−4.86 to 3.93)		*p* = 0.80 F = 0.22 df = 2
2 Months	9.15 ± 4.26			
4 Months	8.00 ± 4.92			
**CSI**				
Baseline	36.62 ± 11.84	−5.15 (−19.42 to 9.11)		*p* = 0.67 F = 0.41 df = 2
2 Months	34.15 ± 14.98			
4 Months	31.46 ± 16.27			

* The mean difference is significant at *p* < 0.001 between baseline and 2 months. *** The mean difference is significant at *p* < 0.001 between baseline and 4 months. PDI—Pain Disability Index; BPIi—Brief Pain Inventory; BPIs—Brief Pain Inventory short form; CSI—Central Sensitization Inventory.

**Table 3:** Comparison of LM, ES, and PS CSA in control and LBP groups measured in cm^2^, adjusted for BMI.

Variables	Measurement Period	Control	LBP
**L3/L4 CSA** **LM**		***n* = 15**	***n* = 12**
	Baseline	6.41 ± 1.43	6.93 ± 1.48
	2 months	6.77 ± 1.41	7.08 ± 1.44
	4 months	6.60 ± 1.50	6.82 ± 1.39
	MD (95% CI)	0.20 (−0.17 to 0.56)	−0.11 (−0.52 to 0.30)
	**Main effect of time**	**Main effect of group**	**Time × group interaction**
	*p* = 0.19 F = 1.75 df = 2	*p* = 0.41 F = 0.70 df = 1	*p* = 0.06 F = 2.91 df = 2
**L3/L4 CSA** **ES**		***n* = 15**	***n* = 12**
	Baseline	18.23 ± 3.91	21.88 ± 5.98
	2 months	18.13 ± 3.77	21.86 ± 5.62
	4 months	18.52 ± 3.62	22.28 ± 5.72
	MD (95% CI)	0.29 (−0.36 to 0.93)	0.40 (−0.32 to 1.12)
	**Main effect of time**	**Main effect of group**	**Time × group interaction**
	*p* = 0.63 F = 0.47 df = 2	*p* = 0.80 F = 0.06 df = 1	*p* = 0.82 F = 0.20 df = 2
**L3/L4 CSA** **PS**		***n* = 9**	***n* = 10**
	Baseline	10.67 ± 2.37	14.46 ± 2.92
	2 months	10.36 ± 2.11	14.19 ± 2.37
	4 months	10.18 ± 2.02	14.11 ± 2.79
	MD (95% CI)	−0.58 (−1.39 to 0.12)	−0.27 (−0.93 to 0.40)
	**Main effect of time**	**Main effect of group**	**Time × group interaction**
	*p* = 0.53 F = 0.65 df = 2	*p* = 0.02 F = 6.34 df = 1	*p* = 0.63 F = 0.11 df = 2
**L4/L5 CSA** **LM**		***n* = 16**	***n* = 12**
	Baseline	9.26 ± 1.72	9.39 ± 1.66
	2 months	9.49 ± 1.88	9.59 ± 1.61
	4 months	9.58 ± 1.83	9.39 ± 1.55
	MD (95% CI)	0.32 (−0.17 to 0.80)	−0.01 (−0.57 to 0.56)
	**Main effect of time**	**Main effect of group**	**Time × group interaction**
	*p* = 0.38 F = 0.97 df = 1.81	*p* = 0.08 F = 3.37 df = 1	*p* = 0.43 F = 0.83 df = 1.81
**L4/L5 CSA** **ES**		***n* = 16**	***n* = 12**
	Baseline	16.10 ± 3.12	18.25 ± 4.28
	2 months	16.13 ± 2.54	18.13 ± 3.82
	4 months	15.97 ± 2.95	18.10 ± 4.45
	MD (95% CI)	−0.13 (−1.03 to 0.78)	−0.15 (−1.21 to 0.91)
	**Main effect of time**	**Main effect of group**	**Time × group interaction**
	*p* = 0.97 F = 0.04 df = 2	*p* = 0.63 F = 0.24 df = 1	*p* = 0.98 F = 0.02 df = 2
**^ǂ^ L4/L5 CSA** **PS**		***n* = 16**	***n* = 10**
	Baseline	16.43 ± 5.38	18.66 ± 3.69
	2 months	16.50 ± 5.27	18.77 ± 3.32
	4 months	15.99 ± 5.04 **	18.33 ± 3.49
	MD (95% CI)	−0.44 (−0.98 to 0.09)	−0.31 (−0.95 to 0.33)
		**Main effect of time**	
		*p* = 0.02 Χ^2^ = 7.88 df = 2	*p* = 0.15 Χ^2^ = 3.80 df = 2
**L5/S1 CSA** **LM**		***n* = 8**	***n* = 6**
	Baseline	11.31 ± 2.57	11.35 ± 1.81
	2 months	11.35 ± 2.30	12.05 ± 2.19
	4 months	11.40 ± 2.68	11.75 ± 2.33
	MD (95% CI)	0.09 (−0.71 to 0.88)	0.40 (−0.52 to 1.32)
	**Main effect of time**	**Main effect of group**	**Time × group interaction**
	*p* = 0.14 F = 2.18 df = 2	*p* = 0.27 F = 1.33 df = 1	*p* = 0.22 F = 1.63 df = 2
**L5/S1 CSA** **ES**		***n* = 8**	***n* = 6**
	Baseline	10.27 ± 2.42	10.66 ± 5.21
	2 months	9.10 ± 2.10	11.82 ± 5.61
	4 months	9.14 ± 1.97	11.22 ± 5.54
	MD (95% CI)	−1.13 (−2.96 to 0.69)	0.56 (−1.54 to 2.67)
	**Main effect of time**	**Main effect of group**	**Time × group interaction**
	*p* = 0.19 F = 1.79 df = 2	*p* = 0.62 F = 0.26 df = 1	*p* = 0.06 F = 3.33 df = 2

** The mean difference is significant at the 0.05 level between 2 months and 4 months. ^ǂ^ Friedman calculation completed for PS CSA L4/L5. CSA—cross-sectional area; LBP—low back pain; LM—lumbar multifidus; ES—erector spinae; PS—psoas.

**Table 4:** Comparison of LM, ES, and PS fat signal fraction in control and LBP groups, adjusted for BMI.

Variables	Measurement Period	Control	LBP
**L3/L4 FSF** **LM**		***n* = 15**	***n* = 12**
	Baseline	24.84 ± 8.71	27.45 ± 11.65
	2 months	25.18 ± 9.01	27.47 ± 11.59
	4 months	24.55 ± 8.06	27.13 ± 12.39
	MD (95% CI)	−0.30 (−1.68 to 1.09)	−0.33 (−1.88 to 1.22)
	**Main effect of time**	**Main effect of group**	**Time × group interaction**
	*p* = 0.47 F = 0.77 df = 2	*p* = 0.82 F = 0.50 df = 1	*p* = 0.76 F = 0.27 df = 2
**L3/L4 FSF** **ES**		***n* = 15**	***n* = 12**
	Baseline	24.74 ± 6.77	28.20 ± 11.91
	2 months	24.74 ± 6.84	27.98 ± 10.32
	4 months	24.53 ± 6.49	27.45 ± 11.85
	MD (95% CI)	−0.21 (−1.61 to 1.19)	−0.75 (−2.31 to 0.82)
	**Main effect of time**	**Main effect of group**	**Time × group interaction**
	*p* = 0.13 F = 2.16 df = 2	*p* = 0.49 F = 0.49 df = 1	*p* = 0.64 F = 0.44 df = 2
**L3/L4 FSF** **PS**		***n* = 9**	***n* = 10**
	Baseline	16.53 ± 2.80	17.21 ± 3.77
	2 months	16.05 ± 2.00	16.30 ± 3.36
	4 months	15.42 ± 2.06 ***	16.29 ± 3.09
	MD (95% CI)	−1.55 (−3.03 to −0.08)	−0.52 (−1.91 to 0.88)
	**Main effect of time**	**Main effect of group**	**Time × group interaction**
	*p* = 0.07 F = 2.87 df = 2	*p* = 0.12 F = 2.70 df = 1	*p* = 0.39 F = 0.98 df = 2
**L4/L5 FSF** **LM**		***n* = 16**	***n* = 12**
	Baseline	27.08 ± 8.28	28.50 ± 10.91
	2 months	27.26 ± 7.94	28.59 ± 11.10
	4 months	27.42 ± 7.65	28.33 ± 11.58
	MD (95% CI)	0.39 (−0.78 to 1.56)	−0.24 (−1.61 to 1.13)
	**Main effect of time**	**Main effect of group**	**Time × group interaction**
	*p* = 0.57 F = 0.57 df = 2	*p* = 0.51 F = 0.45 df = 1	*p* = 0.64 F = 0.45 df = 2
**L4/L5 FSF** **ES**		***n* = 16**	***n* = 12**
	Baseline	30.66 ± 8.73	33.00 ± 13.06
	2 months	30.31 ± 7.89	31.91 ± 11.62
	4 months	31.95 ± 8.44 **	32.55 ± 12.08
	MD (95% CI)	1.19 (−0.52 to 2.90)	−0.30 (−2.30 to 1.70)
	**Main effect of time**	**Main effect of group**	**Time × group interaction**
	*p* = 0.77 F = 0.26 df = 2	*p* = 0.36 F = 0.86 df = 1	*p* = 0.29 F = 1.29 df = 2
**L4/L5 FSF** **PS**		***n* = 16**	***n* = 10**
	Baseline	18.52 ± 3.80	18.54 ± 4.48
	2 months	18.40 ± 4.11	18.64 ± 3.90
	4 months	18.14 ± 2.83	18.49 ± 4.69
	MD (95% CI)	−0.36 (−1.38 to 0.66)	−0.08 (−1.40 to 1.25)
	**Main effect of time**	**Main effect of group**	**Time × group interaction**
	*p* = 0.93 F = 0.07 df = 2	*p* = 0.06 F = 4.10 df = 1	*p* = 0.92 F = 0.09 df = 2
**L5/S1 FSF** **LM**		***n* = 8**	***n* = 6**
	Baseline	29.72 ± 7.48	24.16 ± 4.46
	2 months	29.23 ± 7.83	25.25 ± 5.16
	4 months	29.15 ± 7.55	25.76 ± 5.52
	MD (95% CI)	−0.57 (−2.01 to 0.86)	1.60 (−0.06 to 3.25)
	**Main effect of time**	**Main effect of group**	**Time × group interaction**
	*p* = 0.98 F = 0.02 df = 2	*p* = 0.21 F = 1.79 df = 1	*p* = 0.06 F = 3.15 df = 2
**L5/S1 FSF** **ES**		***n* = 8**	***n* = 6**
	Baseline	36.31 ± 7.81	33.89 ± 12.11
	2 months	35.29 ± 8.33 *	33.27 ± 11.94
	4 months	36.43 ± 8.28	31.86 ± 10.06
	MD (95% CI)	0.13 (−2.60 to 2.85)	−2.03 (−5.17 to 1.11)
	**Main effect of time**	**Main effect of group**	**Time × group interaction**
	*p* = 0.20 F = 1.82 df = 1.17	*p* = 0.10 F = 3.22 df = 1	*p* = 0.41 F = 0.80 df = 1.17

* The mean difference is significant at the 0.05 level between baseline and 2 months. ** The mean difference is significant at the 0.05 level between 2 months and 4 months. *** The mean difference is significant at the 0.05 level between baseline and 4 months. FSF—fat signal fraction; LBP—low back pain; LM—lumbar multifidus; ES—erector spinae; PS—psoas.

**Table 5:** Comparison of LM, ES, and PS CSA % asymmetry in control and LBP groups, adjusted for BMI.

Variables	Measurement Period	Control	LBP
**L3/L4 CSA % asymmetry** **LM**		***n* = 15**	***n* = 12**
	Baseline	5.85 ± 4.06	6.74 ± 3.76
	2 months	8.97 ± 4.37	7.69 ± 5.04
	4 months	6.82 ± 4.85	7.23 ± 3.92
	MD (95% CI)	0.97 (−2.62 to 4.57)	0.49 (−3.53 to 4.51)
	**Main effect of time**	**Main effect of group**	**Time × group interaction**
	*p* = 0.55 F = 0.61 df = 2	*p* = 0.59 F = 0.30 df = 1	*p* = 0.81 F = 0.22 df = 2
**L3/L4 CSA % asymmetry** **ES**		***n* = 15**	***n* = 12**
	Baseline	3.08 ± 2.26	8.23 ± 2.46
	2 months	3.63 ± 2.40	6.93 ± 5.44
	4 months	4.01 ± 2.13	7.22 ± 4.07
	MD (95% CI)	0.93 (−1.16 to 3.01)	−1.01 (−3.35 to 1.32)
	**Main effect of time**	**Main effect of group**	**Time × group interaction**
	*p* = 0.97 F = 0.03 df = 1.86	*p* = <0.001 F = 16.68 df = 1	*p* = 0.47 F = 0.74 df = 1.86
**L3/L4 CSA % asymmetry** **PS**		***n* = 9**	***n* = 10**
	Baseline	8.03 ± 7.35	6.53 ± 3.13
	2 months	5.18 ± 5.12	5.30 ± 3.99
	4 months	5.89 ± 5.19	6.68 ± 6.00
	MD (95% CI)	−2.30 (−9.11 to 4.51)	0.30 (−6.12 to 6.73)
	**Main effect of time**	**Main effect of group**	**Time × group interaction**
	*p* = 0.98 F = 0.03 df = 2	*p* = 0.95 F = 0.01 df = 1	*p* = 0.72 F = 0.33 df = 2
**L4/L5 CSA % asymmetry** **LM**		***n* = 16**	***n* = 12**
	Baseline	5.61 ± 4.73	4.95 ± 3.82
	2 months	5.81 ± 4.00	7.02 ± 6.74 *
	4 months	5.85 ± 3.68	5.63 ± 4.63
	MD (95% CI)	−0.29 (−2.98 to 2.41)	1.38 (−1.79 to 4.54)
	**Main effect of time**	**Main effect of group**	**Time × group interaction**
	*p* = 0.03 F = 3.78 df = 2	*p* = 0.76 F = 0.10 df = 1	*p* = 0.09 F = 2.53 df = 2
**L4/L5 CSA % asymmetry** **ES**		***n* = 16**	***n* = 12**
	Baseline	8.58 ± 5.26	6.15 ± 4.08
	2 months	6.47 ± 4.28	5.34 ± 3.78
	4 months	7.17 ± 5.37	6.17 ± 3.63
	MD (95% CI)	−1.41 (−5.67 t 2.85)	−0.90 (−6.10 to 4.31)
	**Main effect of time**	**Main effect of group**	**Time × group interaction**
	*p* = 0.32 F = 1.16 df = 2	*p* = 0.11 F = 2.79 df = 1	*p* = 0.98 F = 0.02 df = 2
**L4/L5 CSA % asymmetry** **PS**		***n* = 16**	***n* = 10**
	Baseline	7.82 ± 5.28	8.17 ± 4.73
	2 months	8.15 ± 5.51	5.84 ± 3.47
	4 months	6.15 ± 4.22	6.85 ± 4.25
	MD (95% CI)	−1.78 (−4.93 to 1.36)	−1.15 (−5.24 to 2.94)
	**Main effect of time**	**Main effect of group**	**Time × group interaction**
	*p* = 0.81 F = 0.21 df = 2	*p* = 0.65 F = 0.21 df = 1	*p* = 0.42 F = 0.88 df = 2
**L5/S1 CSA % asymmetry** **LM**		***n* = 8**	***n* = 6**
	Baseline	6.21 ± 5.87	7.57 ± 4.83
	2 months	7.15 ± 4.56	6.89 ± 5.25
	4 months	7.14 ± 2.56	5.30 ± 5.65
	MD (95% CI)	0.93 (−4.36 to 6.21)	−2.28 (−8.38 to 3.83)
	**Main effect of time**	**Main effect of group**	**Time × group interaction**
	*p* = 0.99 F = 0.01 df = 2	*p* = 0.70 F = 0.16 df = 1	*p* = 0.61 F = 0.51 df = 2
**L5/S1 CSA % asymmetry** **ES**		***n* = 8**	***n* = 6**
	Baseline	20.37 ± 18.35	17.68 ± 13.84
	2 months	17.92 ± 19.38	17.85 ± 10.33
	4 months	17.83 ± 15.79	18.54 ± 10.54
	MD (95% CI)	−2.55 (−14.72 to 9.63)	0.77 (−13.29 to 14.83)
	**Main effect of time**	**Main effect of group**	**Time × group interaction**
	*p* = 0.73 F = 0.32 df = 2	*p* = 0.32 F = 1.09 df = 1	*p* = 0.74 F = 0.31 df = 2

* The mean difference is significant at the 0.01 level between baseline and 2 months. CSA—cross-sectional area; LBP—low back pain; LM—lumbar multifidus; ES—erector spinae; PS—psoas.

## Data Availability

The data presented in this study are available on request from the corresponding author.
